# Encapsulated *Streptococcus suis* impairs optimal neutrophil functions which are not rescued by priming with colony-stimulating factors

**DOI:** 10.1371/journal.pone.0296844

**Published:** 2024-01-23

**Authors:** Marêva Bleuzé, Jean-Pierre Lavoie, Christian Bédard, Marcelo Gottschalk, Mariela Segura

**Affiliations:** 1 Faculty of Veterinary Medicine, Research Group on Infectious Diseases in Production Animals (GREMIP) & Swine and Poultry Infectious Diseases Research Center (CRIPA), Université de Montréal, St-Hyacinthe, Quebec, Canada; 2 Faculty of Veterinary Medicine, Department of Clinical Sciences, Université de Montréal, St-Hyacinthe, Quebec, Canada; 3 Faculty of Veterinary Medicine, Department of Pathology and Microbiology, Université de Montréal, St-Hyacinthe, Quebec, Canada; University of Auckland, NEW ZEALAND

## Abstract

The porcine pathogen and zoonotic agent *Streptococcus suis* induces an exacerbated inflammation in the infected hosts that leads to sepsis, meningitis, and sudden death. Several virulence factors were described for *S*. *suis* of which the capsular polysaccharide (CPS) conceals it from the immune system, and the suilysin exhibits cytotoxic activity. Although neutrophils are recruited rapidly upon *S*. *suis* infection, their microbicidal functions appear to be poorly activated against the bacteria. However, during disease, the inflammatory environment could promote neutrophil activation as mediators such as the granulocyte colony-stimulating factor granulocyte (G-CSF) and the granulocyte-macrophages colony-stimulating factor (GM-CSF) prime neutrophils and enhance their responsiveness to bacterial detection. Thus, we hypothesized that CPS and suilysin prevent an efficient activation of neutrophils by *S*. *suis*, but that G-CSF and GM-CSF rescue neutrophil activation, leading to *S*. *suis* elimination. We evaluated the functions of porcine neutrophils in *vitro* in response to *S*. *suis* and investigated the role of the CPS and suilysin on cell activation using isogenic mutants of the bacteria. We also studied the influence of G-CSF and GM-CSF on neutrophil response to *S*. *suis* by priming the cells with recombinant proteins. Our study confirmed that CPS prevents *S*. *suis*-induced activation of most neutrophil functions but participates in the release of neutrophil-extracellular traps (NETs). Priming with G-CSF did not influence cell activation, but GM-CSF strongly promote IL-8 release, indicating its involvement in immunomodulation. However, priming did not enhance microbicidal functions. Studying the interaction between *S*. *suis* and neutrophils–first responders in host defense–remains fundamental to understand the immunopathogenesis of the infection and to develop therapeutical strategies related to neutrophils’ defense against this bacterium.

## Introduction

*Streptococcus suis* is a bacterial pathogen affecting pigs and an emerging zoonotic agent. It causes important economic loss to the swine industry due to meningitis, arthritis, endocarditis, septicemia, and sudden death in post-weaned piglets [1]. When *S*. *suis* invades the host, it bypasses immune defenses in the blood and spreads throughout the organism [2,3]. In response to dissemination, neutrophils are quickly recruited, and cytokines are produced in an attempt to control the infection [3–5].

Neutrophils eliminate pathogens using four major effector functions: phagocytosis, degranulation, reactive oxygen species (ROS) production, and neutrophil extracellular traps (NETs) release [6–8]. During phagocytosis, neutrophils engulf the pathogen for intracellular destruction, while during degranulation, they release proteases and enzymes of the cytoplasmic granules to destroy the bacteria present in the microenvironment [7,9]. The membrane enzyme nicotinamide adenine dinucleotide phosphate (NADPH) oxidase transforms the oxygen in ROS (e.g. superoxide anion, hydrogen peroxide, and hydroxyl radicals) highly toxic for the bacteria [7,9]. Activated cells also extrudate DNA fibers covered with histones and granular proteins—structures called NETs–that can capture and kill pathogens [10,11]. The sum of the microbicidal effects of neutrophils is designated as killing. Neutrophils also produce cytokines to orchestrate an appropriate immune response towards infection [12]. These cells play a key role in immune response since their depletion results in a worsened outcome of infectious diseases, as observed during *S*. *suis* infection [13–15].

The full activation of neutrophils requires two steps [16]. First, the “priming” which enhances responsiveness to a second stimulus but does not itself strongly activate the cells [17]. Among the priming agents, granulocyte colony-stimulating factor (G-CSF) and granulocyte-macrophage colony-stimulating factor (GM-CSF) regulate neutrophils at several levels and functions [18,19]. Most studies used “chemical” agonists as a second step of activation (or activation agent) [18–26], while only few studies evaluated the effect of priming on the response to pathogens [27–32].

Despite the arsenal of neutrophils to eliminate pathogens, *S*. *suis* can survive their assault *in vivo* and induce pathology. Virulence factors could help *S*. *suis* thwart host defenses. Among them, the capsular polysaccharide (CPS) is considered a main bacterial virulence factor [33]. Based on its immunogenicity, strains of *S*. *suis* can be divided into 29 serotypes, of which serotype 2 prevails in terms of virulence and prevalence [34]. This thick polysaccharide layer surrounds the bacterium and could hide it from the immune system. *S*. *suis* also secretes a hemolytic toxin, called suilysin, that exerts cytotoxicity on cells and modulates the immune response against the pathogen [33,35]. The microbicidal functions of porcine neutrophils against *S*. *suis in vitro* have recently been reviewed [36], but the literature poorly documents the role of the CPS and the suilysin on the activation of neutrophil functions by *S*. *suis*. Albeit both virulence factors were reported to protect bacteria against phagocytosis and/or killing [36–38], few reports evidenced their role on other functions. We hypothesized that the CPS and suilysin allow *S*. *suis* to modulate multiple neutrophil microbicidal functions, promoting bacterial survival. As aforementioned, the priming of neutrophils could drive a better activation in response to the pathogen and enhance the microbicidal effect. However, the role of neutrophil priming by G-CSF or GM-CSF against *S*. *suis* remains understudied. While no information exists on G-CSF, Chabot-Roy *et al*. [37] reported that GM-CSF primes porcine neutrophil for *S*. *suis* killing. Thus, we speculated that priming with G-CSF or GM-CSF increases neutrophil activation in response to *S*. *suis* despite the protection provided by its virulence factors. Therefore, herein we studied *in vitro* the function of porcine neutrophils in response to *S*. *suis* and determined: (1) the role of CPS and suilysin in the protection of *S*. *suis* against the microbicidal functions of neutrophils; (2) the effect of G-CSF and GM-CSF as priming agents to enhance the responsiveness of cells against this pathogen.

## Materials and methods

### *S*. *suis* serotype 2 strains and growth conditions

The *S*. *suis* strain P1/7 used in the study was isolated from the blood of a pig with meningitis in Europe. The features of the wild-type strain and its derived isogenic mutant are listed in Table 1. The different strains were grown in Todd Hewitt broth (THB; Becton Dickinson, Mississauga, ON, Canada) as previously described [39], then diluted in culture medium before their use for cell activation. We determined the bacterial concentration (CFU/mL) in the final suspension by plating on THB agar.

**Table 1:** *Streptococcus suis* serotype 2 strains used in this study.

Strain	General characteristics	Reference
P1/7	Virulent European strain isolated from a case of pig meningitis in the United Kingdom.	[40]
P1/7Δ*cpsF*	Isogenic non-encapsulated mutant derived from P1/7; in frame deletion of *cpsF* gene.	[41]
P1/7Δ*sly*	Isogenic mutant strain derived from P1/7; in frame deletion of *sly* gene. Deficient for suilysin production.	[42]

### Isolation of porcine blood neutrophils and ethical statement

All experiments involving animals were conducted in accordance with the guidelines and policies of the Canadian Council on Animal Care and the principles set forth in the Guide for the Care and Use of Laboratory Animals by the Animal Welfare Committee of the Université de Montréal (protocol number Rech-2014). Three-week-old, Landrace/white mixed breed weaned piglets were acquired from a commercial farm in Quebec (Canada) with no history of endemic clinical problems caused by *S*. *suis*, no vaccination program against this pathogen, and free of Porcine Reproductive and Respiratory Syndrome virus. The herd was also free of enzootic pneumonia due to *Mycoplasma hyopneumoniae* and clinical disease related to porcine circovirus. Piglets were fed commercial, pelleted non-medicated food, with an addition of dry veggie supplements. Blood of 16 piglets was used throughout the study. For each experiment, the cells came from at least 3 different piglets.

Blood was aseptically collected from the jugular vein (between 5–8 weeks of age) in EDTA coated tubes (Becton Dickinson, Mississauga, ON, Canada), and red cells were sedimented by adding dextran 6% in the tubes for 30 min. The superior fraction (containing neutrophils) was mixed with an equal volume of balanced salt solution and layered on Ficoll-Paque Premium 1,084 (Cytiva, Vancouver, BC, Canada) to undergo density gradient centrifugation for 40 min at 400 *g* at room temperature. The upper layers were discarded and the red cells in the pellet were lysed with pure sterile water. After being washed with a solution of PBS supplemented with 0.5% of bovine serum albumin and 2mM of EDTA, neutrophils were counted and suspended at the appropriate concentration in the suitable medium, depending on the experiment. The purity was above 92% as determined by microscopy using Wright-Giemsa staining (S1 Fig), and viability above 98% as determined by propidium iodide staining method using the ADAM Cell Counter (Montreal Biotech inc., Montreal, Canada).

### Priming of neutrophils

Priming of neutrophils was performed as described elsewhere [37]. We firstly determined the optimal source and concentration of G-CSF by priming the cells with 5, 10, 50, 100 or 200 ng/mL of recombinant G-CSF from different sources: recombinant human G-CSF (Gibco, Burlington, ON, Canada), recombinant human G-CSF (R&D Systems, Toronto, ON, Canada) and recombinant porcine G-CSF (Raybiotech, Burlington, ON, Canada)(S5 Fig). We also included priming with recombinant porcine GM-CSF (R&D Systems, Toronto, ON, Canada). Regarding porcine GM-CSF, a concentration of 50 ng/mL induced neutrophil priming for IL-8 production (see results), suggesting that this concentration is sufficient to prime the cells. Therefore, for the experiments, seeded neutrophils were primed for 30 min at 37°C, 5% CO_2_, with 50 ng/mL of recombinant porcine G-CSF (Raybiotech) or recombinant porcine GM-CSF (R&D Systems), or culture medium for the non-treated group. At the end of the incubation period, primed cells were immediately used for the experiments.

### Neutrophil-mediated killing assay

Untreated neutrophils or primed cells (when indicated) were suspended in a complete medium composed of RPMI 1640 supplemented with 2 mM of L-glutamine and 10 mM of HEPES (all from Gibco) supplemented with 10% of adsorbed and inactivated naïve pig serum (to remove anti-*S*. *suis* antibodies and complement, respectively). Serum adsorption was performed as previously described [43]. A volume containing 1 x 10^6^ cells was placed in microtubes and mixed with bacterial strains P1/7, P1/7 *ΔcpsF* or P1/7 *Δsly* at a multiplicity of infection (MOI) of 1 (final volume 200 μl), as previously described [44,45]. Tubes without neutrophils were prepared in parallel and served to calculate the percentage of killing. The cap of the tubes was pierced to allow oxygenation, and tubes were incubated for 90 min at 37°C, 5% CO_2_ as previously described [37,38]. Tubes were manually mixed every 20 min to ensure contact between cells and bacteria. At the end of the experiment, tubes were vortexted, plated on agar plates and incubated 16 h at 37°C, 5% of CO_2_ for viable count. The percentage of killing was determined using the following formula:

$$\%\;Bacteria\;killed=\left[1-\frac{CFU\;in\;sample}{CFU\;in\;sample\;without\;neutrophils}\right]\;\times 100\%$$

### Neutrophil activation and cytokine quantification

Unprimed or primed neutrophils (when indicated) were suspended in complete medium and plated in 24-well plates (1 x 10^6^ cells/well). Cells were then stimulated with *S*. *suis* (MOI of 1), *Escherichia coli*-purified lipopolysaccharide (LPS) (100 ng/mL; Sigma-Aldrich, Oakville, ON, Canada) as positive control or medium (negative control) for 12 h at 37°C, 5% CO_2_. Since *S*. *suis* becomes cytotoxic for the cells after 12 h of incubation (S2 Fig), 5 000 U/mL of penicillin/streptomycin was added in the wells at 6 h post-stimulation to avoid bacterial-induced cytotoxicity (S3 Fig). At the end of the stimulation, the plate was centrifuged 10 min, 400 *g* at 4°C, the supernatant collected and stocked at– 80°C for further analysis. Levels of cytokines in cell culture supernatants were measured by sandwich ELISA using pair-matched antibodies from R&D Systems, according to the manufacturer’s recommendations, as previously described [46].

### Cytotoxicity assay

Unprimed neutrophils resuspended in complete medium were plated in 24-well plates (1 x 10^6^ cells/well). Cells were then stimulated with *S*. *suis* (MOI of 1), LPS (100 ng/mL) or medium for 4 h, 8 h and 12 h at 37°C, 5% CO_2_, with or without addition of antibiotics at 4 h or 6 h after stimulation. Fresh supernatant was collected, and the cytotoxicity was measured using CytoTox 96^®^ Non-Radioactive Cytotoxicity Assay (Promega, Madison, WI, USA) following manufacturer’s recommendations (S2–S4 Figs).

### Neutrophil extracellular traps (NETs) quantification

NETs quantification was performed as previously described [47]. Briefly, neutrophils were suspended in complete medium (without phenol red). A volume containing 1 x 10^6^ cells was seeded in a 48-well plate and primed as described above or non-treated (control). After priming, cells were stimulated with *S*. *suis* (MOI 1 and 10), or in phorbol 12-myristate 13-acetate (PMA) (1 μM; Invivogen, Burlington, ON, Canada) and LPS (2 μg/mL) for different times at 37°C, 5% CO_2_ [48,49], as positive controls. After incubation, 100 μl of supernatant were collected and put in a black 96-well plate where free double strain DNA was measured using Quant-iT PicoGreen (Invitrogen, Burlington, ON, Canada) following manufacturer’s instructions. The fluorescence was read using the Biotek Synergy LX (Agilent Technologies, Mississauga, ON, Canada) at an excitation wavelength of 480 nm and emission wavelength of 520 nm.

### Visualization of NETs

Three sterile coverslips were put in the bottom of each well of a 6-well plate and coated with freshly collected pig serum by adding 50 μl/coverslip for about 30–60 min at 37°C. Purified neutrophils were suspended 30 min in RPMI 1640 without phenol red supplemented with 2 mM L-glutamine, 50 U/mL Penicillin/Streptomycin, 10% of inactivated fetal bovine serum (FBS) and 20 μM of Hoechst 33342 (Invitrogen). After washing, cells were resuspended in complete RPMI medium without phenol red supplemented with 2% of inactivated FBS. A volume containing 1 x 10^6^ cells was distributed in each well of the 6-well plate containing coverslips and stimulated with *S*. *suis* (MOI of 10) in a medium containing 200 nM of SytoxGreen (Invitrogen). After 2 h of incubation, cells were fixed by adding paraformaldehyde at a final concentration of 4% for 15 min. Each coverslip was rinsed, mounted on a microscope slide, and stored overnight at 4°C in the dark. Samples were observed with an Olympus FluoView^™^ FV1000 confocal laser scanning microscope and analyzed using Fluoview software (Markham, ON, Canada). The fluorescence of Hoechst 33342 was read at an excitation wavelength of 350 nm and an emission wavelength of 461 nm. The fluorescence of SytoxGreen was read at an excitation wavelength of 504 nm and an emission wavelength of 523 nm.

### Degranulation

The amount of myeloperoxidase (MPO) released by neutrophils was quantified as already described [50]. Cells were suspended in PBS and 25 μl distributed in a 96-well plate to reach 1.25 x 10^6^ cells/well. Cells were then primed (or mock-treated) and then activated with 125 μl of *S*. *suis* (MOI of 1 or 10), PMA (1 μM) as positive control or PBS as negative control for 60, 120 or 180 min. In parallel, a cell lysate was obtained by adding sterile pure water to 1.25 x 10^6^ cells. After incubation, 50 μl of 3,3’,5,5’ tetramethylbenzidine (TMB) substrate solution (Life Technologies, Burlington, ON, Canada) was added, directly followed by 50 μl of H_2_O_2_ 5 mM. After 2 minutes, 50 μl of H_2_SO_4_ 2M was added to stop the reaction, and the plate was centrifuged 10 min at 600 *g*. A volume of 200 μl of supernatant was then transferred in a transparent 96-well plate for the reading of optical density (OD) using the Biotek Synergy LX reader, at the wavelength of 450 nm. The percentage of MPO released was determined using the following formula:

$$\%\;MPO\;release=\frac{{(OD}_{stimulated}-{OD}_{unstimulated})}{\left({OD}_{cell\;lysate}-{OD}_{unstimulated}\right)}\;\times 100$$

### Reactive oxygen species (ROS) detection

ROS detection was determined as previously described [51]. After isolation, neutrophils were suspended in PBS supplemented with 20 μM of 2’,7’-dichlorodihydrofluorescein diacetate (H_2_DCF-DA; Invitrogen) for 30 min at 37°C, 5% CO_2_. Cells were then washed and suspended in complete RPMI medium (without phenol red) supplemented with 10% of adsorbed and inactivated naïve pig serum. In a black 96-well plate, 1 x 10^6^ neutrophils/well were primed (or non-treated as control) and then stimulated with *S*. *suis* (MOI 1 or 10), or LPS (100 ng/mL) as positive control at different times. The fluorescence was read using the Biotek Synergy LX at an excitation wavelength of 495 nm and emission wavelength of 520 nm.

### Statistical analysis

Bar graphs represent means +/- SEM. Figures depict combined results from at least three independent experiments and SEMs were calculated from combined datasets. To evaluate statistical differences between groups, we performed parametric (unpaired *t*-test) or non-parametric tests (Mann–Whitney rank sum test), when appropriate. Each test was repeated in at least three independent experiments. A *P* value below 0.05 was considered statistically significant.

## Results

### The CPS, but not suilysin, protects *S*. *suis* against killing by neutrophils

**Fig 1:**
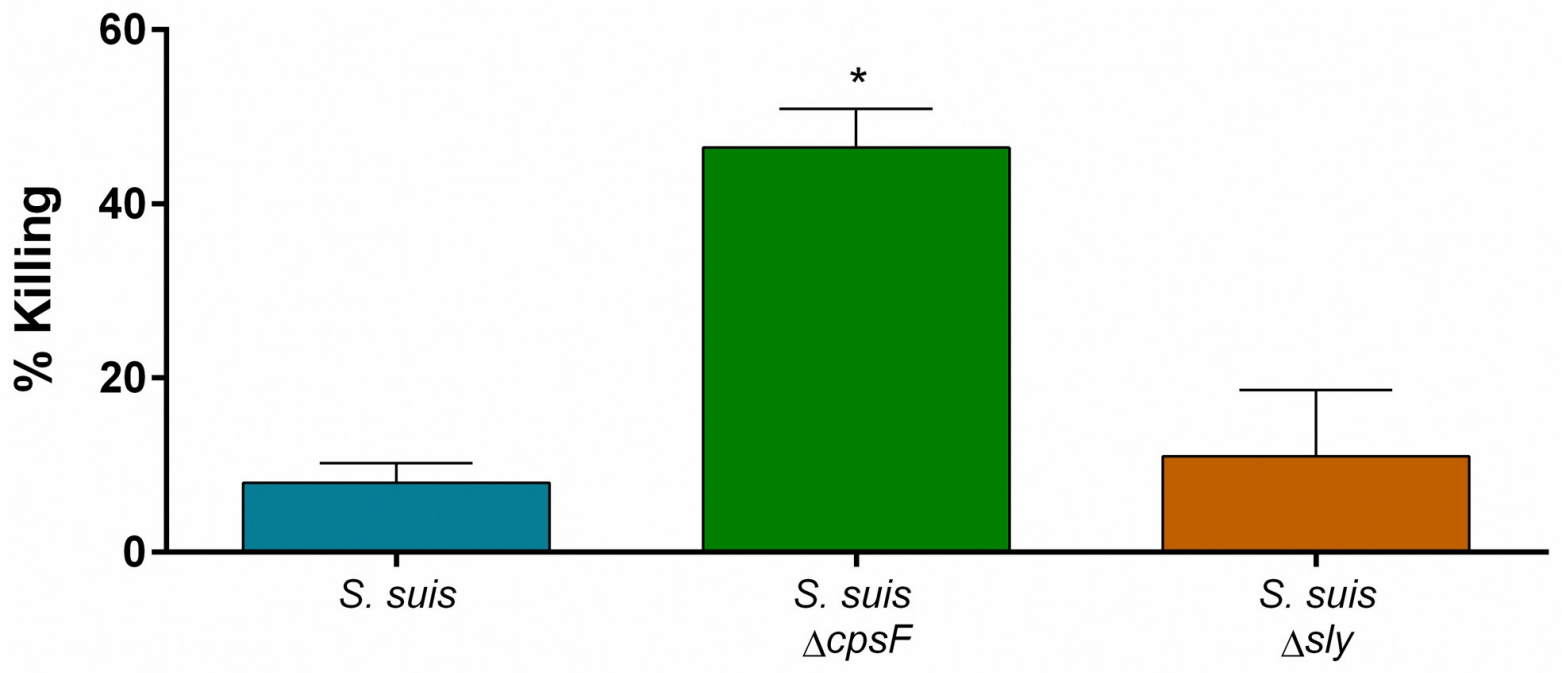
Capsular polysaccharide (CPS) but not suilysin (sly) protects *S*. *suis* from killing mediated by neutrophils. Porcine neutrophils (non-primed) were incubated with *S*. *suis* wild-type strain, its non-encapsulated mutant (*S*. *suis* Δ*cpsF*) and sly-negative mutant (*S*. *suis* Δ*sly*) at a multiplicity of infection (MOI) of 1. After 90 min of incubation, content was plated on agar plates and colony-forming units were counted. Percentage of killing was calculated in comparison to bacterial growth without neutrophils. * indicates a significant difference compared to *S*. *suis* (*P* < 0.001), as determined using the t-test or the Mann-Whitney rank sum test.

Neutrophils possess an important antimicrobial activity against a variety of bacteria [52]. However, their capacity to kill *S*. *suis* remains controversial [36]. To determine the global efficacy of neutrophils to eliminate *S*. *suis* in our model, we performed a killing assay with unprimed cells and revealed that only a low percentage (below 8%) of *S*. *suis* wild-type strain was killed by neutrophils under our experimental conditions (Fig 1). Since *S*. *suis* main virulence factors could participate in its resistance to neutrophil killing capacity [37,38], we performed a killing assay with isogenic mutants deficient for CPS or suilysin expression. We confirmed that, while neutrophils killed suilysin-deficient mutant (*S*. *suis* Δ*sly*) similarly to wild-type strain, they killed significantly more of the non-encapsulated mutant (*S*. *suis* Δ*cpsF*), reaching an average of 46.5% of bactericidal effect (Fig 1).

### CPS, but not suilysin, limits *S*. *suis* induction of IL-8 release by neutrophils

**Fig 2:**
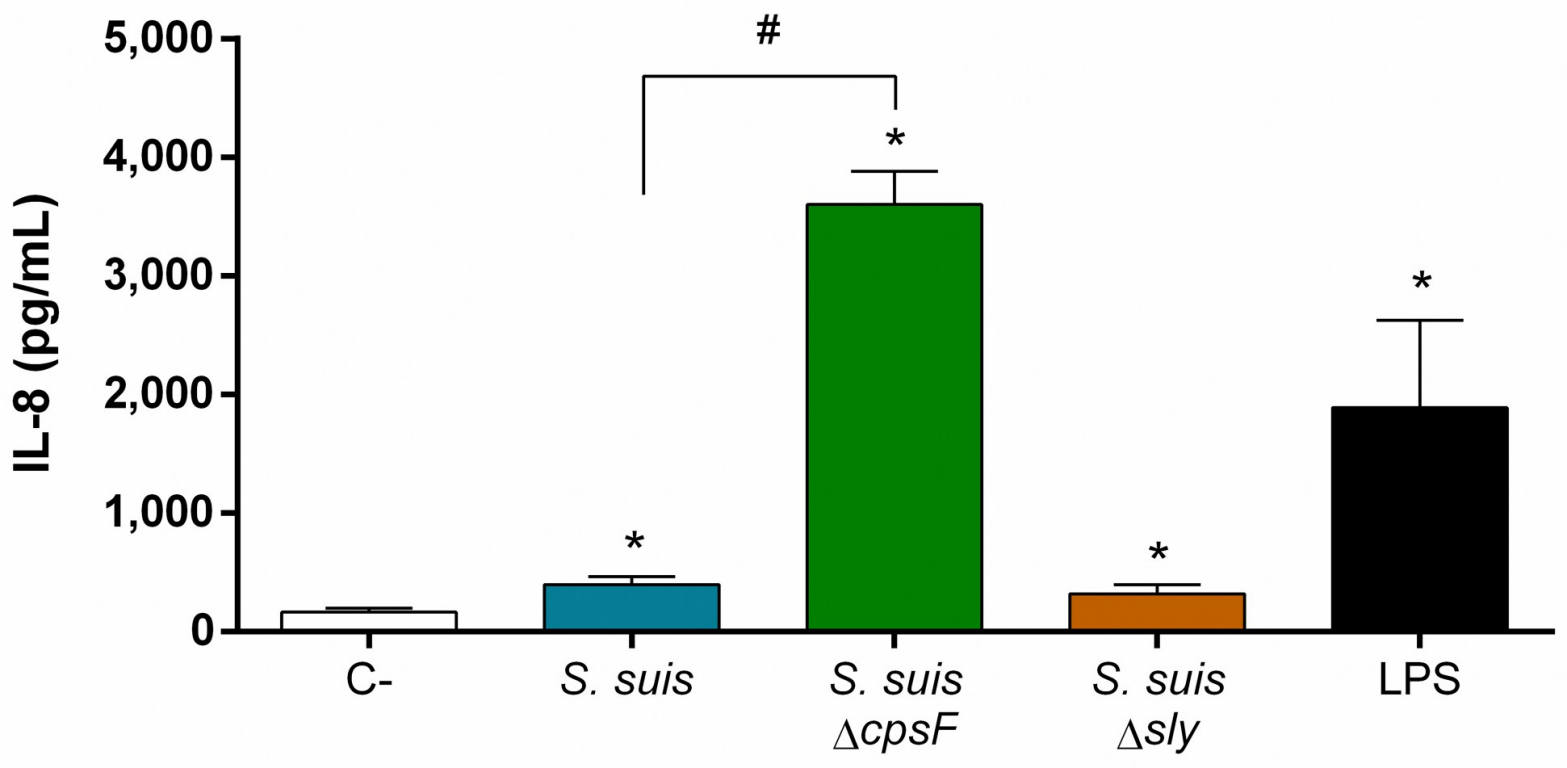
Capsular polysaccharide (CPS) prevents *S*. *suis* recognition by neutrophils and subsequent IL-8 production. Porcine neutrophils (non-primed) were stimulated with *S*. *suis* wild-type strain, its non-encapsulated mutant (*S*. *suis* Δ*cpsF*) or its suilysin (sly)-negative mutant (*S*. *suis* Δ*sly*) at a multiplicity of infection (MOI) of 1, or with positive control lipopolysaccharide (LPS; 100 ng/mL). C- correspond to unstimulated control cells. To prevent cytotoxicity caused by *S*. *suis* multiplication, antibiotics were added after 6 h of incubation. Neutrophils were stimulated for a total of 12 h and supernatant analyzed by ELISA. * indicates a significant difference compared to C- (*P* < 0.05); # indicates a significant difference compared to *S*. *suis* wild-type (*P* < 0.001), as determined using the t-test or the Mann-Whitney rank sum test.

To coordinate immune responses, cells communicate through cytokine production. Neutrophils, like almost every cell type, produce cytokines when they detect microorganisms [12]. To evaluate cytokine production in response to *S*. *suis*, we quantified the IL-8 released by neutrophils in response to various stimuli [53]. *S*. *suis* induced only a slight but significant release of IL-8 by neutrophils (Fig 2). While the suilysin-deficient mutant activated the cells similarly to the wild-type strain, the non-encapsulated mutant induced almost 10 times more releases of IL-8 by neutrophils (Fig 2). Other cytokines were also measured, but porcine neutrophils did not produce IL-6 (12.6 ± 11 pg/mL for *S*. *suis* and 4.4 ± 6 pg/mL for LPS) or TNF-α (under the limit of detection) in response to stimulation. In parallel to IL-8 quantification, we evaluated the cytotoxicity of the two mutant strains towards neutrophils, since the wild-type strain exhibit a strong cytotoxicity after 12 h of stimulation in the absence of antibiotics (S4 Fig). Both strains had a reduced cytotoxic effect on neutrophils. In the case of the non-encapsulated mutant, this observation is likely due to the reduced number of bacteria in the wells (Fig 1).

### The expression of CPS, but not the suilysin, favors NET release by neutrophils in response to *S*. *suis*

**Fig 3:**
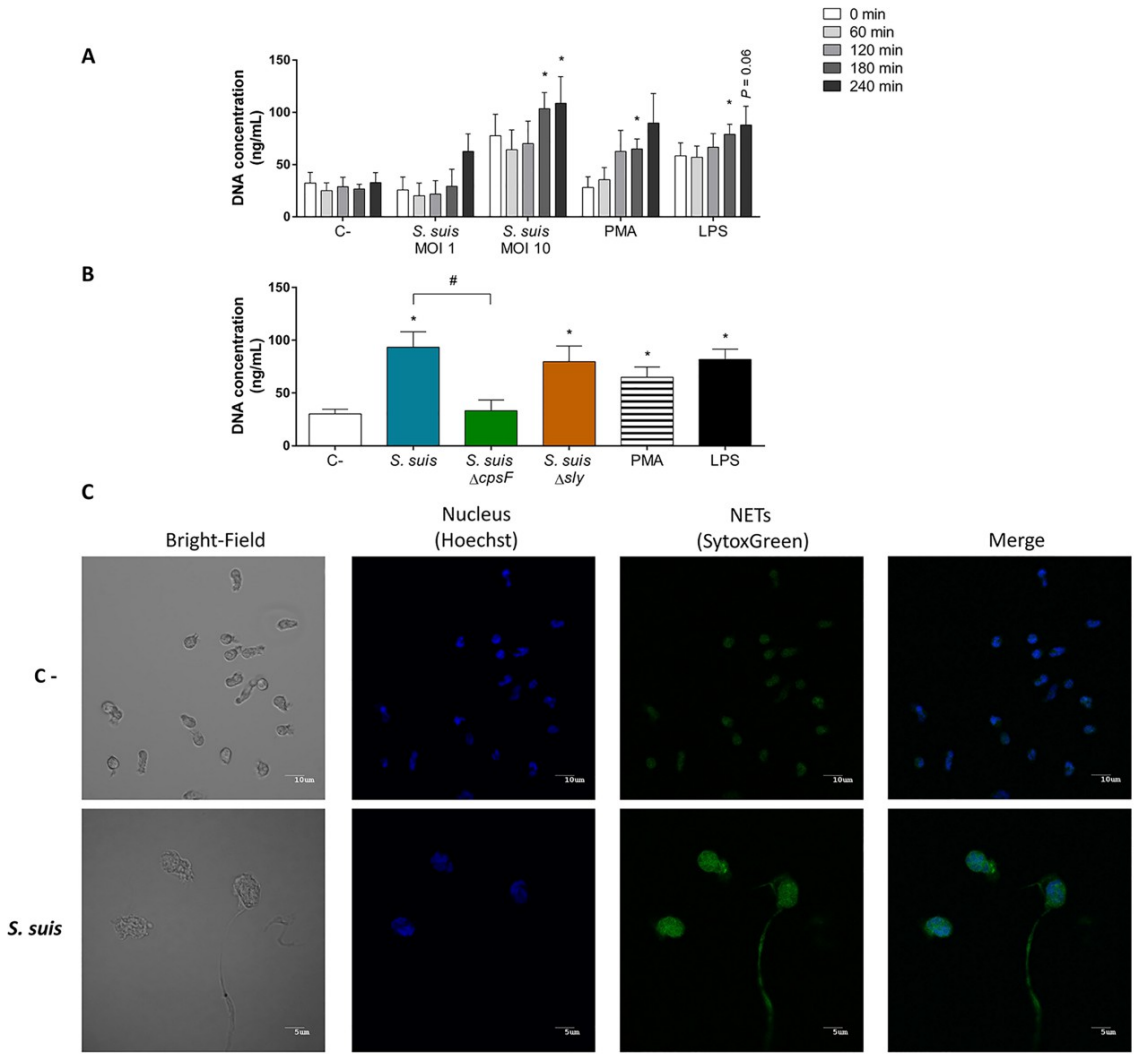
*S*. *suis* induces NET release which is favored by the presence of the capsular polysaccharide (CPS). **(A, B)** Non-primed purified porcine neutrophils were stimulated with *S*. *suis* (at a multiplicity of infection (MOI) of 1 or 10), or positive controls phorbol-12-myristate-13-acetate (PMA; 1 μM) and lipopolysaccharide (LPS; 2 μg/mL). C- designate unstimulated control cells. After stimulation, released double-stranded DNA was measured in the supernatant using Picogreen. **(A)** Kinetics of DNA release by porcine neutrophils stimulated with *S*. *suis*. * represents a significant difference when compared to C- (*P* < 0.05), as determined using the t-test or the Mann-Whitney rank sum test. **(B)** Effect of *S*. *suis* virulence factors on its ability to induce NET release by neutrophils. Neutrophils were stimulated with *S*. *suis*, its non-encapsulated mutant (*S*. *suis* Δ*cpsF*) or its suilysin (sly)-negative mutant (*S*. *suis* Δ*sly*) at a MOI of 10 for 180 min. * represents a significant difference when compared to C- (*P* < 0.01); # represents a significant difference when compared to *S*. *suis* wild-type strain (*P* < 0.01), as determined using the t-test or the Mann-Whitney rank sum test. **(C)** Neutrophiles stimulated with *S*. *suis* release long DNA fibers called NETs. Nucleus of living cells was marked with Hoechst 33342 before seeding in coverslips. Neutrophils (non-primed) were then stimulated with *S*. *suis* wild-type strain (MOI of 10) for 2 h in a medium containing SytoxGreen, a marker of DNA. Images were obtained by confocal microscopy.

Neutrophils can release NETs in response to stimuli. The deployed DNA fibers, covered by proteins, in the neutrophil environment can catch and kill pathogens [10]. To evaluate NET release by *S*. *suis*-stimulated neutrophils, we quantified the free DNA in the cell supernatant. A kinetic study indicated that *S*. *suis*, at a MOI of 10, induces a significant NET release by neutrophils no sooner than 180 min after stimulation with no further increase with longer incubation times (Fig 3A). The structures were observable by confocal microscopy (Fig 3C). However, at a MOI of 1, *S*. *suis* did not induce a significant NET release (Fig 3A). To investigate the role of virulence factors in NET induction, we stimulated cultured cells with the non-encapsulated or suilysin-deficient mutant. The stimulation with the non-encapsulated mutant led to a significant reduction in NET release compared to the wild-type strain (Fig 3B). However, the neutrophils stimulated with the suilysin-deficient mutant released an amount of NETs similar to the wild-type strain (Fig 3B). Surprisingly the positive control PMA, which induces high levels of NETs by mouse and human neutrophils [54,55], had a moderate effect on porcine neutrophils, which was similar to that of LPS, also used as positive control.

### The CPS, but not the suilysin, of *S*. *suis* weakens neutrophil degranulation in response to bacteria

**Fig 4:**
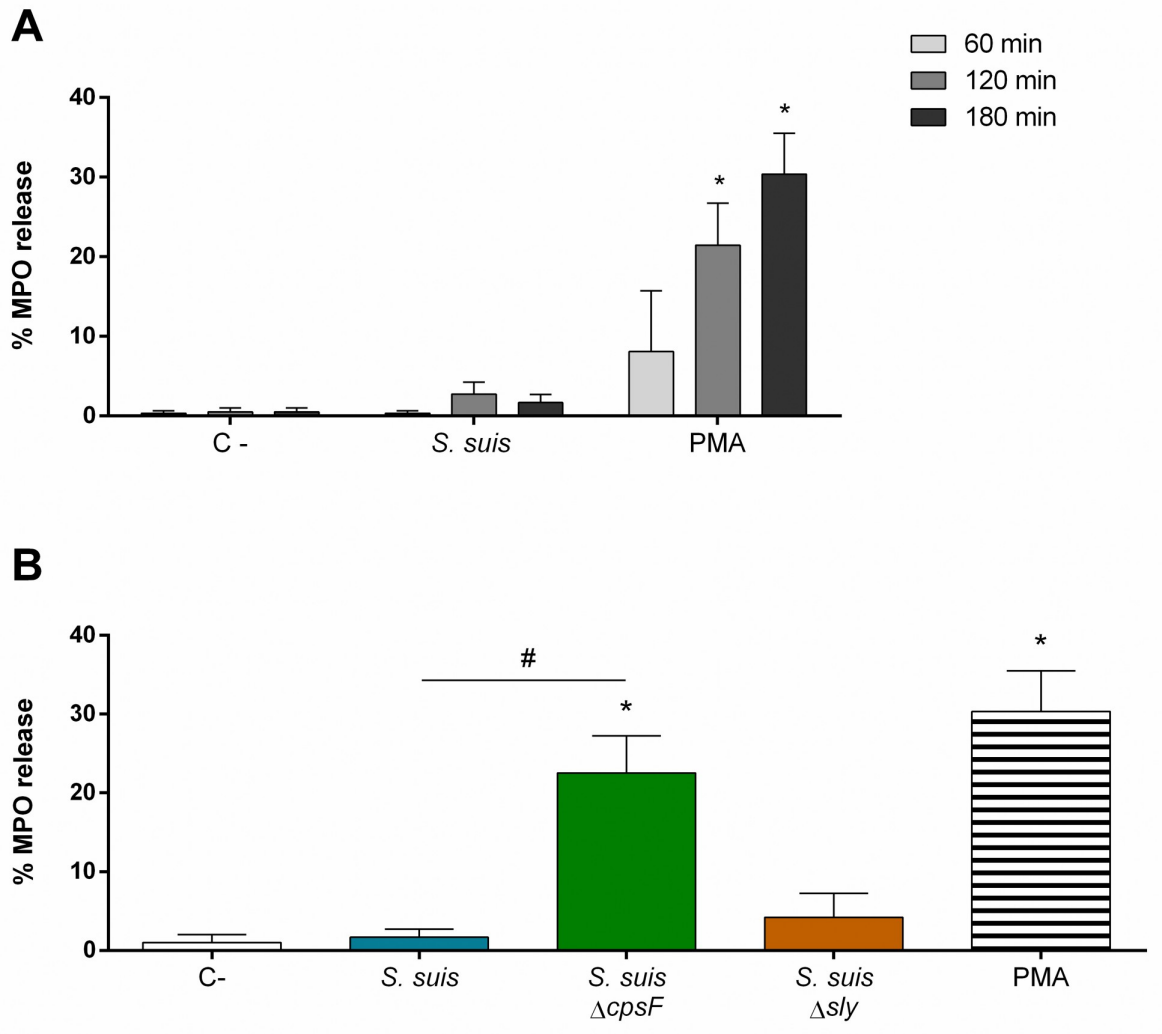
*S*. *suis* capsular polysaccharide (CPS) prevents degranulation of neutrophils exposed to bacteria. Porcine neutrophils (non-primed) were stimulated with *S*. *suis* or controls, and myeloperoxidase (MPO) release evaluated by a colorimetric reaction with 3,3’,5,5’-tetramethylbenzidine hydrochloride (TMB) and oxygen peroxide (H_2_O_2_). **(A)** Degranulation kinetic of *S*. *suis*-stimulated neutrophils. Cells were stimulated with *S*. *suis* wild-type strain at a multiplicity of infection (MOI) of 1 or the positive control phorbol-12-myristate-13-acetate (PMA; 1 μM) for different incubation times. **(B)** Degranulation induced by isogenic mutants of *S*. *suis*. Cells were then stimulated with *S*. *suis*, its non-encapsulated mutant (*S*. *suis* Δ*cpsF*) or its suilysine (sly)-negative mutant (*S*. *suis* Δ*sly*); or positive control PMA (1 μM) for 180 min. * represents a significant difference compare to C- cells stimulated with PBS (*P* < 0.05); # represents a significant difference compare to cells stimulated with *S*. *suis* wild-type strain (*P* < 0.05), as determined using the t-test or the Mann-Whitney rank sum test.

Degranulation refers to the exocytosis of the cytoplasmic granules of the neutrophils. Their content in proteases and enzymes can then destroy bacteria present in the phagosome or in the microenvironment [9]. To evaluate degranulation, we quantified the granule enzyme MPO, released by the cells in response to a strong activator signal [56]. The kinetics of MPO release (which includes soluble or NET associated) induced by *S*. *suis* wild-type strain showed that neutrophils degranulated less than 2% in response to bacteria (MOI of 1) after 180 min of stimulation. At this time-point, the positive control PMA induced 30% of degranulation, indicating that cells can be activated very quickly after stimulation (Fig 4A). A MOI of 10 did not improve the percentage of MPO release by *S*. *suis* -activated neutrophils (0.9 ± 1% at 120 min and 10.3 ± 14% at 180 min of incubation with *S*. *suis* MOI of 10). When we evaluated the influence of the CPS and suilysin on the induction of the degranulation, we revealed that the non-encapsulated mutant triggers a significant increase of MPO release (*P* = 0.02). One the other hand, the suilysin-deficient mutant induced a degranulation similar to that of the wild-type *S*. *suis* (Fig 4B).

### Neutrophils show no modulation of ROS production in response to *S*. *suis*

**Fig 5:**
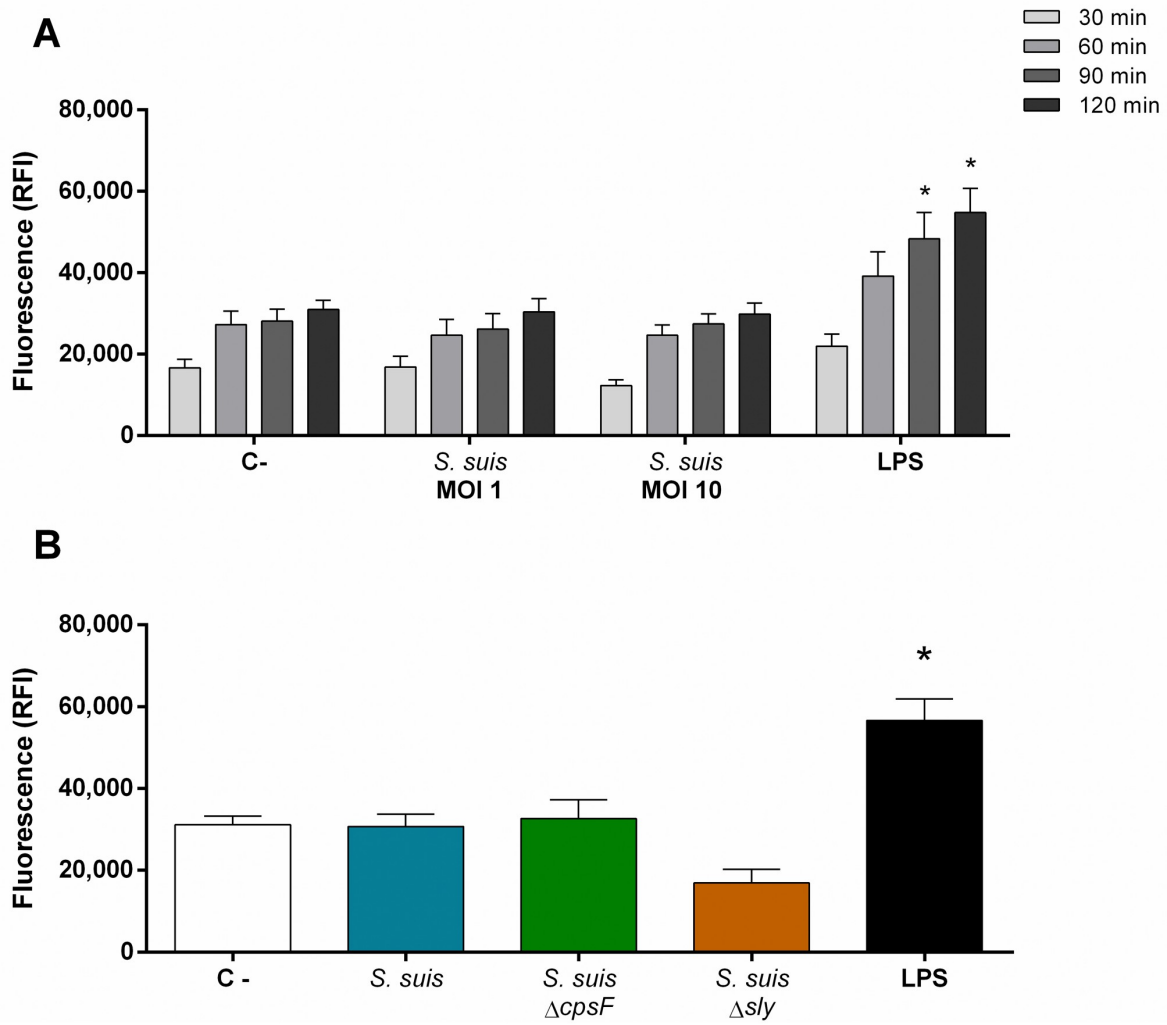
Neutrophils do not produce reactive oxygen species (ROS) when stimulated by *S*. *suis*. Purified porcine neutrophils (non-primed) were plated and stimulated with *S*. *suis* at a multiplicity of infection (MOI) of 1 or 10, or positive control lipopolysaccharide (LPS; 100 ng/mL). C- represents unstimulated control cells. ROS were measured using 2’,7’-dichlorodihydrofluorescein diacetate (H_2_DCF-DA) reactive which turns fluorescent when oxidized. Fluorescence was read after stimulation at an Ex/Em of 495/520 nm. **(A)** ROS production kinetics by stimulated porcine neutrophils. **(B)** Role of *S*. *suis* virulence factors in ROS production by neutrophils. Cells were stimulated with *S*. *suis*, its non-encapsulated mutant (*S*. *suis* Δ*cpsF*) or its suilysin (sly)-negative mutant (*S*. *suis* Δ*sly*) at multiplicity of infection (MOI) of 1; or LPS for 120 min. * represents a significant increase compared to C- cells (*P < 0.05), as determined using the t-test or the Mann-Whitney rank sum test.

As the reactive oxygen species (ROS) damper the growth of *S*. *suis* [57], we investigated if non-primed porcine neutrophils produce ROS in response to *S*. *suis*. Neutrophils did not produce ROS when stimulated with the wild-type bacteria, although they were activated as soon as 90 min of stimulation with the positive control LPS (Fig 5A). When investigating the role of virulence factors, the non-encapsulated mutant and the suilysin-deficient mutant failed to increase ROS production when compared to control non-activated cells (Fig 5B).

### Neutrophils poorly kill *S*. *suis* even after priming with G-CSF or GM-CSF

**Fig 6:**
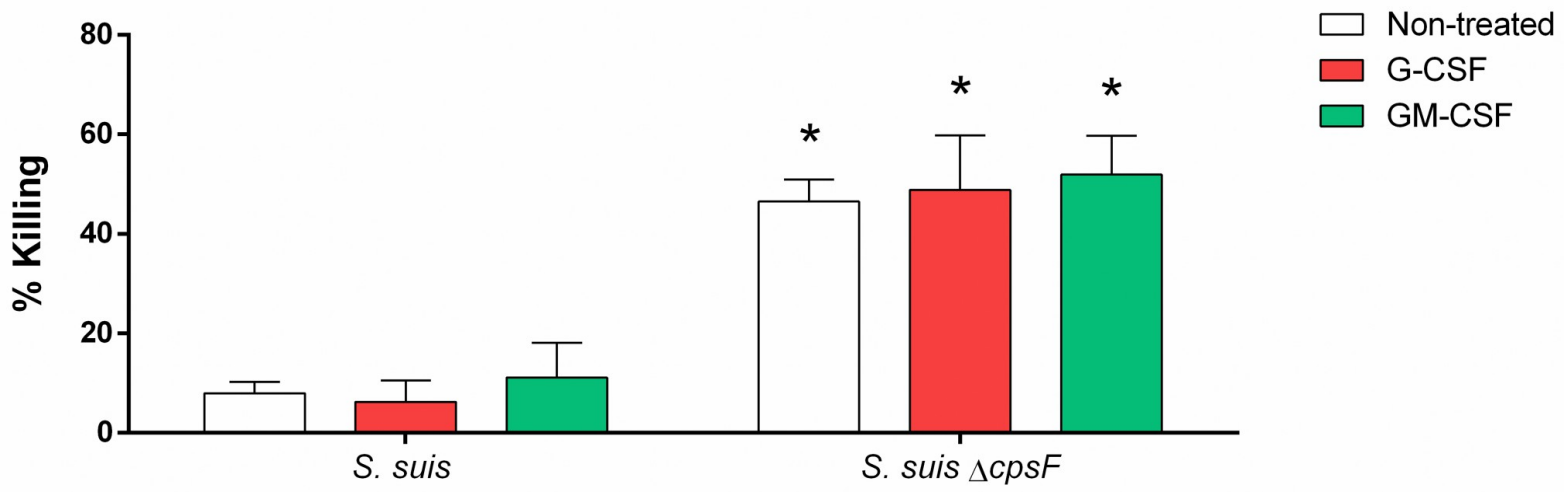
G-CSF and GM-CSF do not promote *S*. *suis* killing mediated by neutrophils. Purified neutrophils were infected with *S*. *suis* wild-type strain or its non-encapsulated mutant (*S*. *suis* Δ*cpsF*) at a multiplicity of infection (MOI) of 1. Before contact, neutrophils were primed with 50 ng/mL of G-CSF or GM-CSF, or left unprimed (non-treated). After 90 min of incubation, the remaining live bacteria were plated on agar plates and colony-forming units were counted. Percentage of killing was calculated in comparison to bacterial growth without neutrophils. * indicates a significant difference compared to *S*. *suis* wild-type (*P* < 0.001), as determined using the t-test or the Mann-Whitney rank sum test.

The priming of neutrophils with growth factors or cytokines could improve the microbiocidal functions of the cells towards pathogens [27,29,31]. We thus investigated whether stimulating neutrophils with the granulocyte colony-stimulating factor (G-CSF) or the granulocyte-macrophage colony-stimulating factor (GM-CSF)–two growth factors essential for neutrophil control—could improve cell efficacy to kill *S*. *suis*. Priming with G-CSF and GM-CSF had no effect on the killing properties of neutrophils. Since neutrophils killed more efficiently the non-encapsulated mutant, we investigated if the priming with G-CSF or GM-CSF could further enhance the killing of non-encapsulated *S*. *suis* by the cells. However, we observed no changes compared to non-treated control cells (Fig 6).

### GM-CSF increases IL-8 production by neutrophils in response to *S*. *suis*

**Fig 7:**
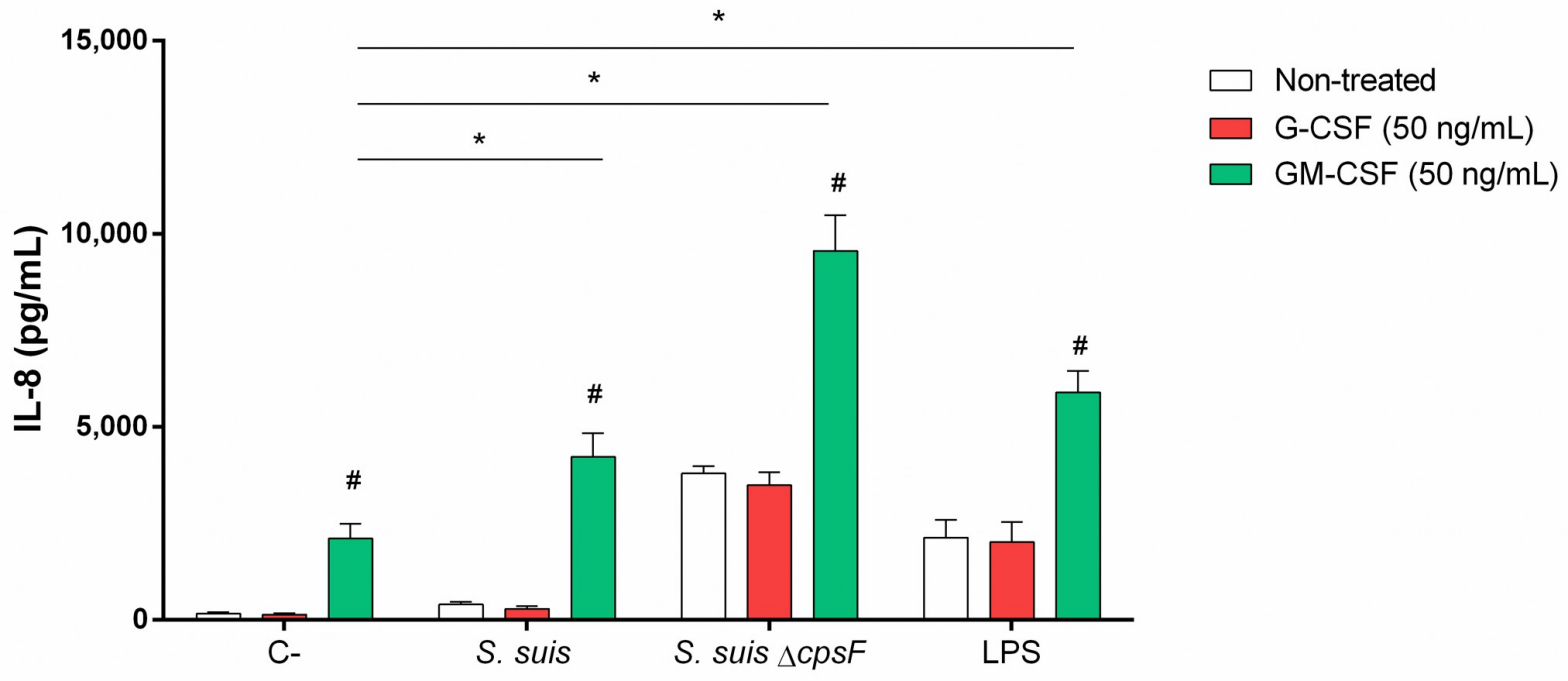
Priming with GM-CSF enhances cytokine production by neutrophils, but priming with G-CSF does not. Porcine neutrophils were non-treated or primed with G-CSF or GM-CSF (50 ng/mL) for 30 min. They were then stimulated with *S*. *suis* or its unencapsulated mutant (*S*. *suis* Δ*cpsF*) at a multiplicity of infection (MOI) of 1; or positive control lipopolysaccharide (LPS; 100 ng/mL). C- correspond to unstimulated control cells. To prevent cytotoxicity caused by *S*. *suis* multiplication, antibiotics were added after 6 h of incubation. Neutrophils were stimulated 12 h and supernatant analysed by ELISA. Non-treated cells stimulated with *S*. *suis* and LPS produced higher IL-8 than unstimulated C- cells (*P* < 0.05 –not indicated). # indicates a significant difference compared to respective non-treated cells (*P* < 0.001), * indicates a significant difference compared to C- (*P* < 0.01), as determined using the t-test or the Mann-Whitney rank sum test.

Among cellular functions that could be affected by priming, the production of cytokines may be increased by treatment with G-CSF or GM-CSF [58]. We thus investigated if priming of porcine neutrophils with G-CSF or GM-CSF enhances IL-8 release in response to *S*. *suis*. The treatment with G-CSF (either at 50, 100 or 200 ng/mL) did not increase the levels of IL-8 released by the cells, whether they were non-stimulated (C-) or stimulated with bacteria or LPS (positive control) (Figs 7 and S5). Treatment with GM-CSF, however, induced a marked increase of released IL-8 levels by neutrophils, whether they were stimulated or not. But we observed a synergistic effect when cells were activated after priming with *S*. *suis*, its unencapsulated mutant or LPS, as IL-8 concentrations greatly exceeded those of cells only primed with GM-CSF.

### Priming with G-CSF or GM-CSF does not influence the release of NETs induced by *S*. *suis*

**Fig 8:**
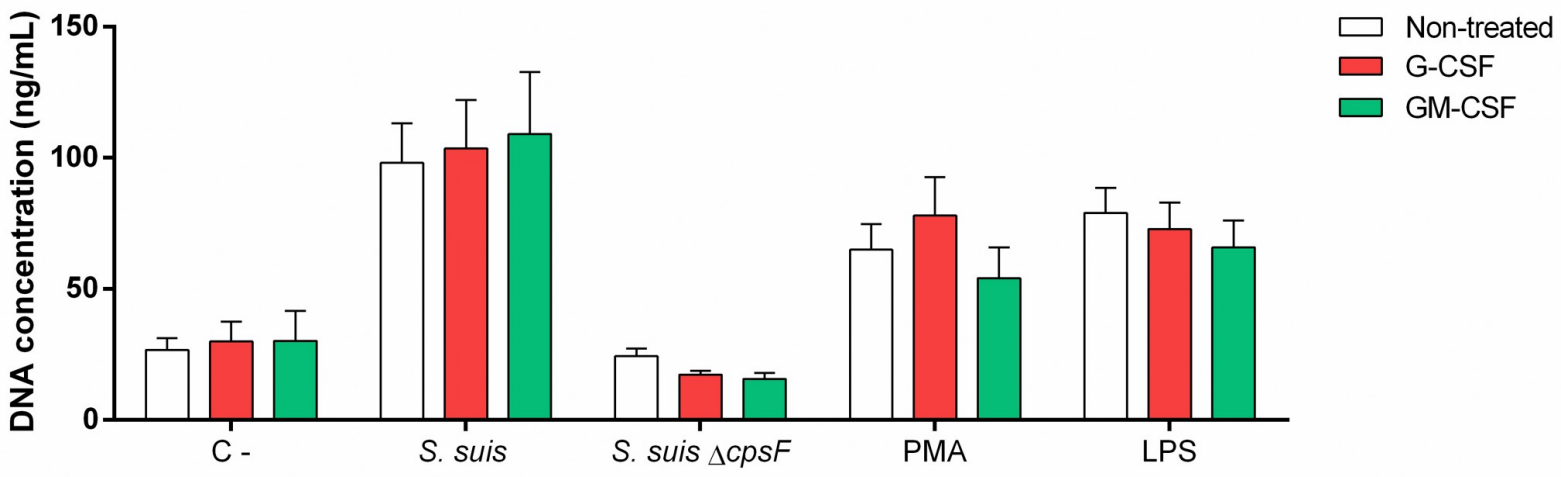
*S*. *suis-*induced NET release by neutrophils is not influenced by priming with G-CSF or GM-CSF. Purified porcine neutrophils were primed 30 min with G-CSF and GM-CSF (50 ng/mL), then stimulated for 180 min with *S*. *suis* or its unencapsulated mutant (*S*. *suis* Δ*cpsF*) at a multiplicity of infection (MOI) of 10; or positive controls phorbol-12-myristate-13-acetate (PMA; 1 μM) and lipopolysaccharide (LPS; 2 μg/mL). C- designate unstimulated control cells. After stimulation, released double-stranded DNA was measured in the supernatant using Picogreen. Difference between C- and *S*. *suis* was significant for all the treatments (*P* < 0.01 –not indicated), as determined using the t-test or the Mann-Whitney rank sum test.

As demonstrated above (Fig 3), the only neutrophil function markedly induced by wild-type *S*. *suis* is NET release. Literature report that both G-CSF and GM-CSF induce NET release by neutrophils [22,59]. We evaluated if this phenomenon can be observed in the context of *S*. *suis* infection. In our model, G-CSF or GM-CSF priming does not affect the amount of DNA liberated by neutrophils in response to *S*. *suis* stimulation (Fig 8).

### G-CSF and GM-CSF do not enhance the degranulation of neutrophils in response to encapsulated *S*. *suis*

**Fig 9:**
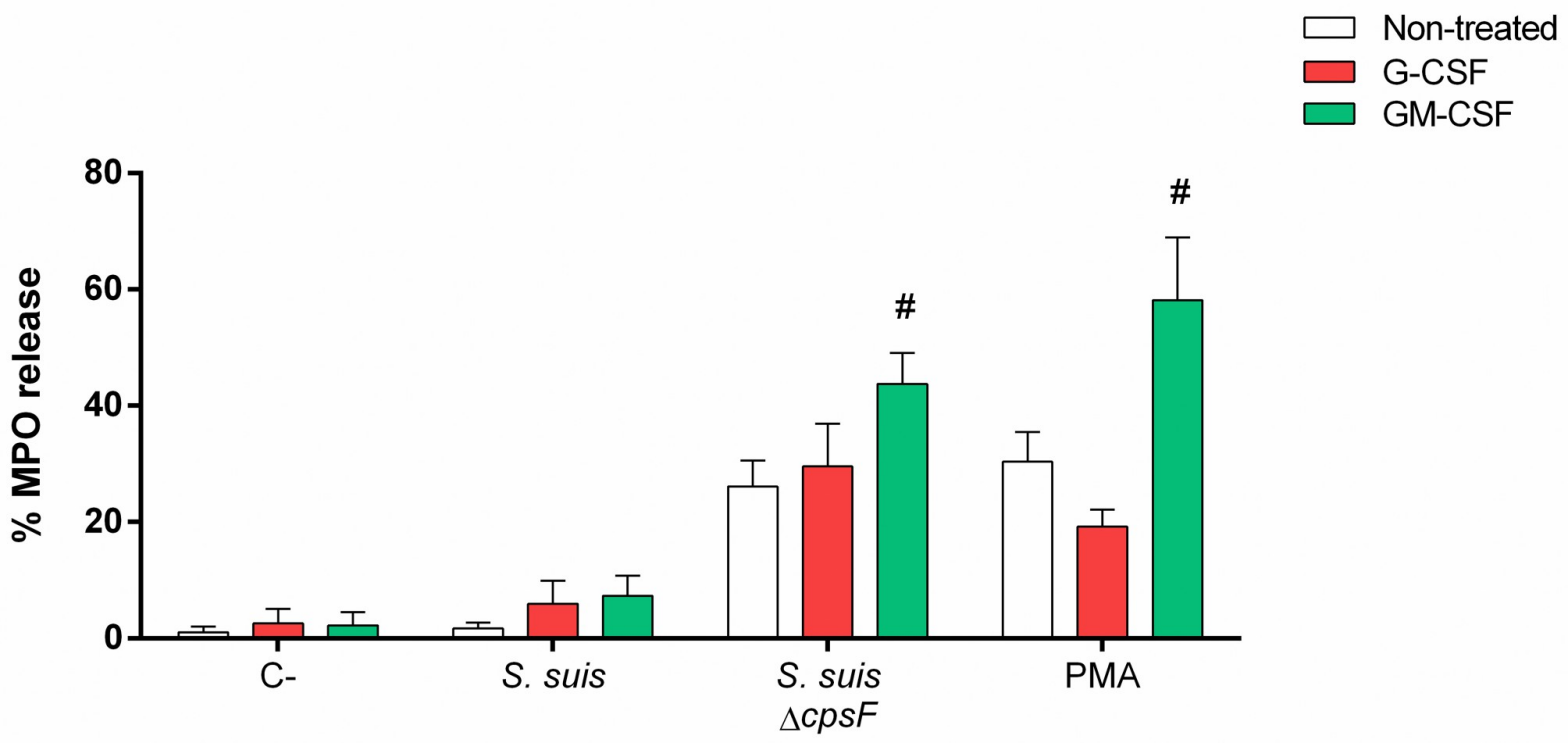
Degranulation of *S*. *suis*-stimulated neutrophils is not improved by priming with G-CSF and GM-CSF. Porcine neutrophils were primed with 50 ng/mL of G-CSF and GM-CSF or left non-treated for 30 min. They were then stimulated with *S*. *suis* or its isogenic mutant (*S*. *suis* Δ*cpsF*) at a multiplicity of infection (MOI) of 1; or positive control (PMA; 1 μM) for 180 min. MPO release was evaluated by a colorimetric reaction with 3,3’,5,5’-tetramethylbenzidine hydrochloride (TMB) and oxygen peroxide (H_2_O_2_). # represents a significant difference compared to respective non-treated cells (*P* < 0.05), as determined using the t-test or the Mann-Whitney rank sum test.

G-CSF and GM-CSF prime degranulation of neutrophils activated by formylmethionyl leucyl phenylalanine (fMLP) [26,60]. We thus investigated if the growth factors could enhance cell degranulation in response to *S*. *suis* by quantifying released MPO. Results showed no changes in the amount of MPO released in presence of *S*. *suis* wild type strain (Fig 9). But interestingly, GM-CSF priming significantly increased the MPO released in presence of the non-encapsulated mutant (*P* = 0.03) and the positive control PMA (Fig 9).

### Neither G-CSF nor GM-CSF influences ROS production by neutrophils stimulated with *S*. *suis*

**Fig 10:**
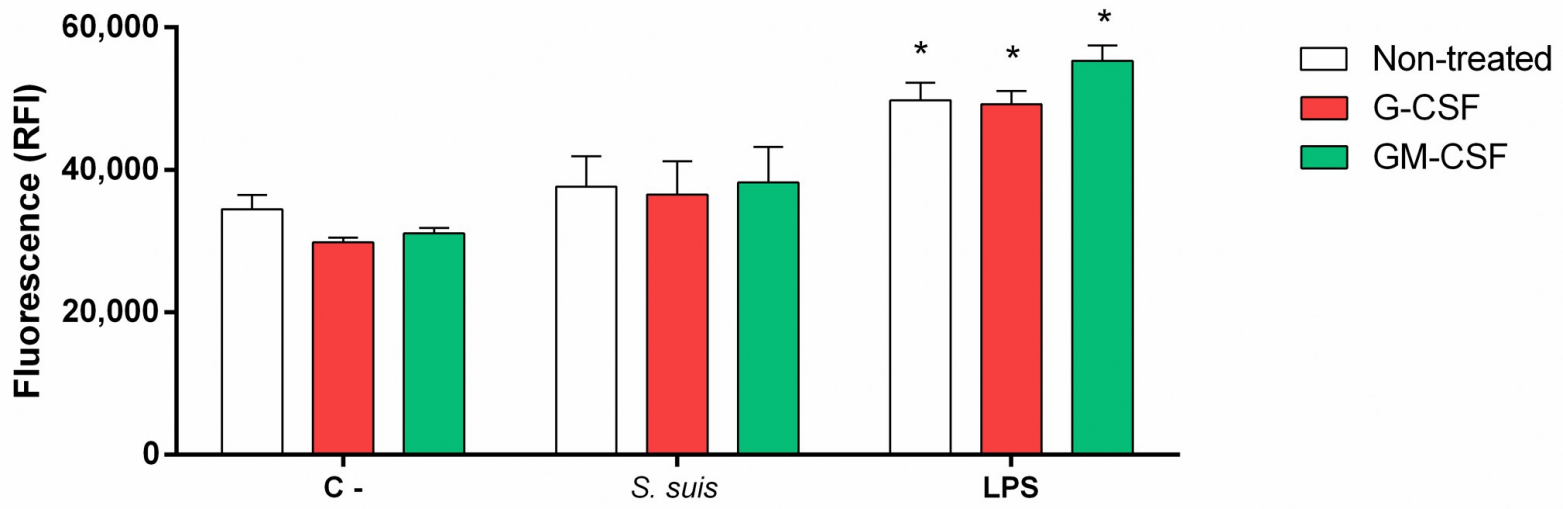
Neutrophils do not produce reactive oxygen species (ROS) when stimulated by *S*. *suis*, even after priming with G-CSF or GM-CSF. Purified porcine neutrophils were primed 30 min with G-CSF or GM-CSF (50 ng/mL) before stimulation with *S*. *suis* at a multiplicity of infection (MOI) of 1 or lipopolysaccharide (LPS; 100 ng/mL) for 120 min. C- represent unstimulated control cells. ROS were measured using 2’,7’-dichlorodihydrofluorescein diacetate (H_2_DCF-DA) reactive which turns fluorescent when oxidized. Fluorescence was read after stimulation at an Ex/Em of 495/520 nm. * represents a significant difference compare to respective C- cells (*P* < 0.001), as determined using the t-test or the Mann-Whitney rank sum test.

As for the other functions, studies describe that G-CSF and GM-CSF prime neutrophils for ROS production [26,61]. However, our results showed no influence of priming with G-CSF or GM-CSF on ROS production by neutrophils stimulated with *S*. *suis* (Fig 10).

## Discussion

Infection with *S*. *suis* in pigs induces a marked recruitment of neutrophils and a systemic release of cytokines, including colony-stimulating factors [3,62]. However, the pathogen bypasses the immune host defenses and disseminates in the host, causing clinical manifestations [1]. In this study, we aimed to understand how *S*. *suis* thwarts neutrophils’ responses by evaluating how it modulates their functions *in vitro*. We also determined if priming with G-CSF or GM-CSF could enhance neutrophil response towards *S*. *suis* and ultimately promotes its elimination.

Albeit several studies have already evaluated the killing of *S*. *suis* by porcine neutrophils, contradictory results emerge in the literature. Our results side with studies reporting a poor killing of *S*. *suis* [37,45,63–68] but differ from those obtained in other studies [57,69–71]. Differences in methodology (multiplicity of infection, presence of serum, etc.) could explain the divergence observed among studies. We confirmed in our model that the CPS greatly participates in *S*. *suis* survival against neutrophil-meditated killing as demonstrated elsewhere [36–38,45,65,72]. Protected by its CPS, the bacteria grow and cause cytotoxic effects on neutrophils through the action of suilysin. Among the possible mechanisms implicated in this resistance, the CPS could hide surface proteins rendering neutrophils unable to detect bacteria properly and prevent phagocytosis by destabilizing lipid microdomains [41,73–76].

Our data revealed for the first time that porcine neutrophils produce low but significant levels of IL-8 (also known as CXCL8) in response to *S*. *suis*. The chemokine IL-8 recruits neutrophils at the infection site by chemotaxis, and it activates their functions, like degranulation and production of ROS [77]. In our model, *S*. *suis*-stimulated neutrophils did not produce TNF-α and IL-6, as reported in another study [46]. We demonstrated that CPS masks surface proteins of the bacteria, which prevents recognition by neutrophils and subsequent release of IL-8. This observation was made for other cell types, such as dendritic cells and macrophages [41,74,76,78–83], and for other cytokines such as TNF-α and IL-10 [46]. It was suggested that activation of immune cells for cytokine production occurs through recognition of subcapsular mature lipoproteins via Toll-like receptor (TLR) 2 [62,81,84]; however, this needs to be confirmed with pig neutrophils. Apart from external recognition, the lack of phagocytosis due to the CPS prevents the intracellular recognition of the microbe and subsequent cytokine production.

Neutrophil stimulation with *S*. *suis* resulted in a significant release of NETs, confirming previous observations [44,68,85,86]. Since it is the only function markedly activated, it might be pivotal in the pathogenesis of *S*. *suis* infection; however, the net beneficial or detrimental effect of NETs needs to be determined. A defect in NET function renders the host susceptible to infections [8]. In the case of *S*. *suis*, even though bacteria resist NET microbicidal activity *in vitro* [68], they could capture *S*. *suis* and prevent its spread. Furthermore, the treatment of *S*. *suis*-infected mice with DNAse–that dismantle NET structures–worsens the outcome of the infection *in vivo* [87]. On the other hand, an excess of NETs may participate in the exacerbation of the inflammation in different contexts. For example, they can induce tissue damage due to associated granular proteins, cause thrombosis in hosts with sepsis, and they correlate with the severity of sepsis in humans [11,88–90]. When we evaluated its role in the induction of NETs by neutrophils, we demonstrated for the first time that CPS participates (directly or indirectly) in the activation of NET release by neutrophils, instead of preventing their activation as observed for other functions. Contradictory observations were made in the literature, as the CPS of *Burkholderia pseudomallei* (Gram-negative bacterium) and of *Cryptococcus neoformans* (fungi) prevent NET release by human neutrophils [91,92], while no role for the CPS was observed for group A *Streptococcus* [93]. It could be hypothesized that *S*. *suis* CPS itself activates NET formation, as reported with the purified CPS of *Streptococcus pneumoniae* [94]. However, this hypothesis remains to be demonstrated for *S*. *suis*. A second hypothesis is that cells slightly detect *S*. *suis* despite the CPS, but being uncapable to ingest *S*. *suis*, they engage NETs as a last resort against bacteria. To form NETs, the neutrophil elastase (NE)–a granular enzyme—needs to be present in the cytosol, but an efficient phagocytosis leads to the sequestration of NE in the phagosome [95,96]. In the case of *S*. *suis*, since CPS prevents phagocytosis, NE could reach the cytosol and trigger NET release by the cells. This could explain why the non-encapsulated mutant do not induce NET release: neutrophils detect and phagocyte non-encapsulated bacteria and engage an appropriate response able to kill the pathogens, abrogating the necessity to activate NET release.

Our study first described that porcine neutrophils poorly degranulate in response to *S*. *suis*, while a study on human cells reported a significant degranulation compared to uninfected neutrophils [97]. To quantify degranulation, we dosed the release of MPO [98], an enzyme of the primary (or azurophilic) granules that necessitates a strong activation signal to be exocytosized [56]. Furthermore, our study demonstrated that the CPS prevents degranulation by neutrophils. Two hypotheses might explain this effect of CPS. First, CPS could mask surface proteins able to activate neutrophil degranulation through TLR, integrins, or G-protein coupled receptors [55,99]. Then, the fact that CPS impedes phagocytosis might consequently prevent the activation of intracellular receptors, such as TLR9 and TLR8, known to be involved in degranulation [100,101].

Our study also showed that *S*. *suis* does not induce the oxidative burst (ROS) of porcine neutrophils, confirming the observation of Rungelrath *et al*. [57]. In the latter study, the oxidative burst was triggered by *S*. *suis* only in the presence of hyperimmune serum. Results with pig neutrophils are inconsistent with those obtained with mouse and human neutrophils, which report an increase of ROS production in *S*. *suis*-stimulated cells, confirming the importance of studying cells from different species [47,51,102]. Compared to other neutrophil functions, CPS was not responsible for the lack of ROS induction by *S*. *suis*. Since ROS production depends on the recognition of bacteria by G-protein coupled receptors, integrins, Fc receptors or TLRs [103], several hypotheses can be emitted. First, the subcapsular component would be unable to activate ROS production by the cells, or unidentified virulence factors could inhibit ROS production. For example, the superoxide dismutase of *S*. *suis* was described as a factor that transforms superoxide anion into less damageable ROS [104]. From the host perspective, ROS production also greatly depends on opsonizing factors, as already suggested [57]. Since our technical approach aimed to reflect the global oxidative response of neutrophils, a more precise characterization of the different ROS could highlight the mechanisms by which *S*. *suis* counteracts activation. Similarly, the role of ROS in *S*. *suis*-induced NETosis remains to be clarified.

*S*. *suis* secretes suilysin, a toxin known to be cytotoxic and immunomodulatory [33,35]. Although suilysin causes cytotoxicity towards porcine neutrophils, our results suggest that it has no major role in the activation or modulation of porcine neutrophil functions. In contrast to other studies, suilysin did not modulate the killing by porcine neutrophils in our model [37,38]. This discrepancy can be explained by the different methods used in these studies. A study by Chen *et al*. [97] corroborated that suilysin does not influence ROS production but pointed out that it induces the degranulation of human neutrophils. Therefore, the role of suilysin in the interactions of *S*. *suis* with neutrophils remains to be further characterized.

The priming of neutrophils with G-CSF and GM-CSF could enhance their activation and facilitate *S*. *suis* elimination. Priming of neutrophils was often conducted with a “chemical” second signal and rarely with whole pathogens. In this study, we evaluated for the first time how the priming of porcine neutrophils with G-CSF and GM-CSF modifies their response toward *S*. *suis*. However, in our model, G-CSF did not prime neutrophils for any of the functions we studied. Reports using whole pathogens found that G-CSF primes for oxidative burst, phagocytosis, and killing [27–30], in neutrophils from species other than pigs. Nevertheless, priming represents only one of the numerous functions of the G-CSF. It could be responsible for neutrophil recruitment from the bone marrow as already described for other inflammatory contexts [18,105]. To corroborate this idea, Brockmeier *et al*. [106] injected G-CSF in pigs and observed an immediate increase in blood neutrophils and delayed mortality associated with *S*. *suis* infection. However, more studies are needed to clarify the role of G-CSF in *S*. *suis* infection.

The priming of porcine neutrophils with GM-CSF revealed various phenomenon. Firstly, GM-CSF did not prime neutrophils for the killing of *S*. *suis* in our model, unlike the results obtained by Chabot-Roy *et al*. [37] that showed that GM-CSF improves the killing function of neutrophils towards *S*. *suis*. As mentioned above, technical details for killing experiments might contribute to variable outcomes. We also evidenced that GM-CSF does not prime for NET release or ROS production, regardless of the second signal (*S*. *suis* or positive control). Conversely, even if GM-CSF does not prime degranulation in response to wild-type *S*. *suis*, it enhances responsiveness to non-encapsulated *S*. *suis* or positive control. Thus, we could reasonably hypothesize that GM-CSF primes neutrophils for degranulation, but CPS prevents full activation of the neutrophils, by hiding surface proteins and/or limiting phagocytosis (which respectively prevent extracellular and intracellular activation of the degranulation). The only function greatly modulated by GM-CSF is the release of IL-8. Indeed, GM-CSF itself enhanced IL-8 release by neutrophils, but it also had a synergic effect when coupled to a second signal, including *S*. *suis* wild-type. IL-8 plays an important role in mounting an appropriate response during infectious diseases; however, the consequence of such an increased production can be detrimental. Indeed, IL-8 allows massive recruitment of immune cells, including neutrophils, whose only possible response against *S*. *suis* is the release of NETs, since CPS prevents optimal activation of the other functions. Indeed, in spite of increased IL-8 release after GM-CSF priming, amplification of ROS production and degranulation in the presence of encapsulated *S*. *suis* was not observed. Furthermore, *S*. *suis* was suggested to survive NET microbicidal effect and escape trapping [54,107]. An excess of NETs can promote the genesis of an exacerbated inflammation. Thus, the partial priming of neutrophils by GM-CSF could ultimately worsen the outcome of the infection.

## Conclusion

**Fig 11:**
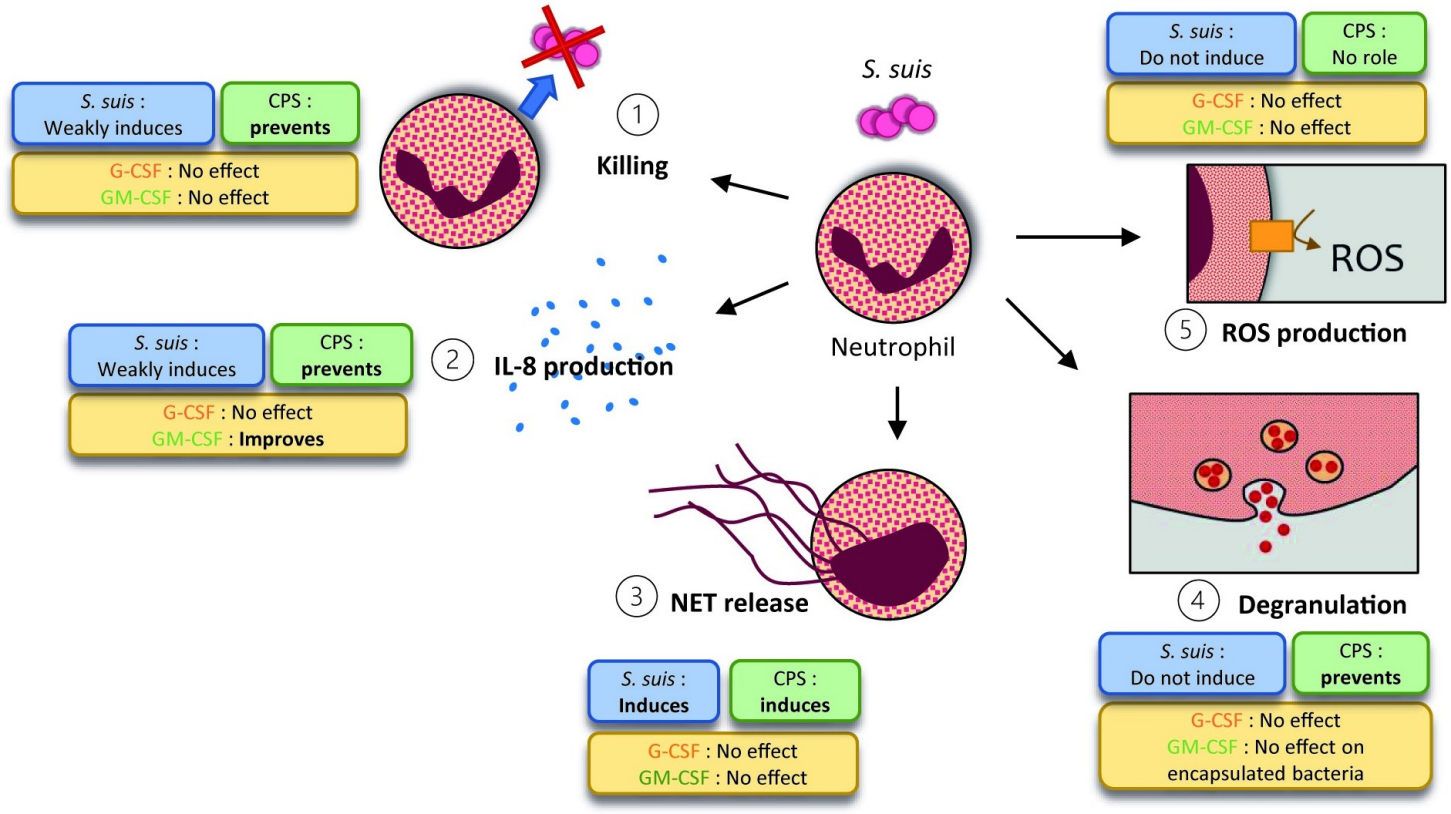
Proposed model for *S*. *suis* activation of porcine neutrophils. The figure depicts how *S*. *suis* activates the five main functions of porcine neutrophils (blue squares), the role of its capsular polysaccharide (CPS) (green squares) and the influence of the priming by the granulocyte colony-stimulating factor (G-CSF) and the granulocyte-macrophages colony-stimulating factor (GM-CSF) (yellow squares). ① Neutrophils weakly kill *S*. *suis* due to its CPS and priming with G-CSF and GM-CSF do not enhance killing. ② *S*. *suis* induces a slight production of IL-8 by neutrophils although CPS prevents full activation. GM-CSF activates and primes neutrophils for IL-8 production. ③ *S*. *suis* induced neutrophil extracellular trap (NET) release by neutrophils which depends on the expression of the CPS. ④ *S*. *suis* do not stimulates neutrophil degranulation since CPS protect bacteria. ⑤ *S*. *suis* do not induce reactive oxygen species (ROS) production by neutrophils but no role can be attributable to the CPS.

The interaction between *S*. *suis* and the immune system remains crucial for understanding how the bacteria cause disease. Given that *S*. *suis* weakly activates neutrophil functions, we hypothesized that bacterial virulence factors prevent cell activation. However, we propose that priming with G-CSF and GM-CSF rescues optimal neutrophil activation. Our results confirm that the subdued activation of neutrophil functions impedes their ability to eliminate *S*. *suis*. (Fig 11). Nonetheless, neutrophils release NETs in response to *S*. *suis*, potentially contributing to exacerbated inflammation. The CPS of *S*. *suis* promotes the bacterium’s survival, preventing neutrophils from degranulating and producing IL-8, thereby altering the initial immune response to the bacteria. Although GM-CSF enhances IL-8 production by neutrophils, neither G-CSF nor GM-CSF improves the bactericidal functions of neutrophils under our experimental conditions. This study describes the neutrophil response to *S*. *suis* using cells from the natural host, focusing on modulation by both bacterial and host factors. It advances our understanding of the early stages of *S*. *suis* immuno-pathogenesis, specifically the interactions between *S*. *suis* and neutrophils. Further research is needed to determine if neutrophils mount an appropriate response towards *S*. *suis in vivo*.

## Supporting information

S1 Fig The purity of porcine neutrophil suspension was measured using two methods. After purification by gradient density, the purity of porcine neutrophil suspension was evaluated using two different methods: a Giemsa-Wright staining followed by manual differential on 100 cells and an automatic cell differential with the Advia 120 hematology analyzer (Siemens Healthcare, Tarrytown, New York, USA).
(PDF)

S2 Fig *S*. *suis* induces a significant production of IL-8 by porcine neutrophils after 12 h of stimulation but present cytotoxic effects. Purified porcine neutrophils were stimulated for different times with *S*. *suis* wild type at various multiplicity of infection (MOI) or with positive control lipopolysaccharide (LPS; 100 ng/mL). C- correspond to unstimulated control cells. (A) The IL-8 in the supernatant was quantified by ELISA. (B) Cytotoxicity was determined by measuring the amounts of lactate dehydrogenase (LDH) in fresh supernatant using a colorimetric reaction. The dotted line represents the threshold of 25% under which the condition is considered non-cytotoxic. * represents a significant difference compared to C- (*P* < 0.05).
(PDF)

S3 Fig *S*. *suis* induces a significant production of IL-8 by porcine neutrophils without cytotoxicity after 12 h of stimulation, when antibiotics are added to the wells after 6 h. Purified porcine neutrophils were stimulated for a total of 12 h with *S*. *suis* wild type at a multiplicity of infection (MOI) of 1. After 4 h or 6 h post-infection (p.i.), 5 000 U/mL of antibiotics (ATB) were added to the cells to control bacteria multiplication. C- correspond to unstimulated control cells. (A) The IL-8 in the supernatant was quantified by ELISA. (B) Cytotoxicity was determined by measuring the amounts of lactate dehydrogenase (LDH) in fresh supernatant using a colorimetric reaction. The dotted line represents the threshold of 25% under which the condition is considered non-cytotoxic. * represents a significant difference compared to C- (*P* < 0.05).
(PDF)

S4 Fig The cytotoxicity induced by *S*. *suis* on porcine neutrophils after 12h depends on the expression of the capsular polysaccharide (CPS) and the suilysin. Purified porcine neutrophils were stimulated for 12 h with *S*. *suis* wild type, *S*. *suis* Δ*cpsF*, *S*. *suis* Δ*sly* at a multiplicity of infection (MOI) of 1. After incubation, the supernatant was collected and the lactate dehydrogenase (LDH) measured by a colorimetric reaction. The LDH is released by lysed cells and its amount reflects the percentage of cytotoxicity. The dotted line represents the threshold of 25% under which the condition is considered non-cytotoxic. * represents a significant difference (*P* < 0.05).
(PDF)

S5 Fig Dose-response of G-CSF treatment on IL-8 production by porcine neutrophils. Porcine neutrophils were non-treated or primed with G-CSF (5, 10, 50, 100 or 200 ng/mL) for 30 min. They were then stimulated with *S*. *suis* at a multiplicity of infection (MOI) of 1; or positive control lipopolysaccharide (LPS; 100 ng/mL). C- correspond to unstimulated control cells. To prevent cytotoxicity caused by *S*. *suis* multiplication, antibiotics were added after 6 h of incubation. Neutrophils were stimulated 12 h and supernatant analysed by ELISA. Non-treated cells stimulated with *S*. *suis* and LPS produced higher IL-8 than unstimulated C- cells (*P* < 0.05 –not indicated). The statistical analyses were performed using the t-test or the Mann-Whitney rank sum test.
(PDF)

S1 Appendix (XLSX)

## Abbreviations

CFU: colony-forming unit
CPS: capsular polysaccharide
CXCL: chemokine (C-X-C motif) ligand
DNA: deoxyribonucleic acid
EDTA: ethylenediaminetetraacetic acid
ELISA: enzyme-linked immunosorbent assay
FBS: fetal bovine serum
fMLP: formylmethionyl leucyl phenylalanine
G-CSF: granulocyte colony-stimulating
GM-CSF: granulocyte-macrophage colony-stimulating factor
H_2_DCF-DA: 2′,7′-dichlorofluorescin diacetate
H_2_O_2_: hydrogen peroxide
H_2_SO_4_: sulfuric acid
HEPES: N-2-hydroxyethylpiperazine-N’-2-ethanesulfonic acid
IL: interleukin
LPS: lipopolysaccharide
MOI: multiplicity of infection
MPO: myeloperoxidase
NADPH: nicotinamide adenine dinucleotide phosphate
NE: neutrophil elastase
NET: neutrophil extracellular trap
PBS: phosphate-buffered saline
PMA: phorbol 12-myristate 13-acetate
ROS: reactive oxygen species
RPMI: Roswell Park Memorial Institute
SEM: standard error of the mean
sly: suilysine
THB: Todd Hewitt broth
TLR: Toll-like receptor
TMB: 3,3’,5,5’ tetramethylbenzidine
TNF: tumor necrosis factor
WT: wild-type
