# Supplementary figures and images for: Encapsulated Streptococcus suis impairs optimal neutrophil functions which are not rescued by priming with colony-stimulating factors
Source: PLoS One. 2024 Jan 23;19(1):e0296844. doi: 10.1371/journal.pone.0296844 (PMC10805302; doi:10.1371/journal.pone.0296844)

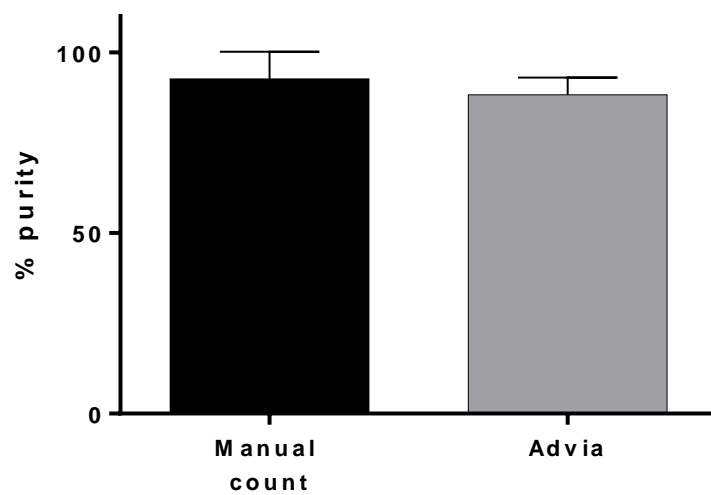

|                | Manual count | Advia |
|----------------|--------------|-------|
| Mean           | 92.65        | 88.20 |
| Std. Deviation | 7.509        | 4.770 |

Supplement: S1 Fig — After purification by gradient density, the purity of porcine neutrophil suspension was evaluated using two different methods: a Giemsa-Wright staining followed by manual differential on 100 cells and an automatic cell differential with the Advia 120 hematology analyzer (Siemens Healthcare, Tarrytown, New York, USA). (PDF) [file pone.0296844.s001.pdf]

**A****Cytokine production kinetics**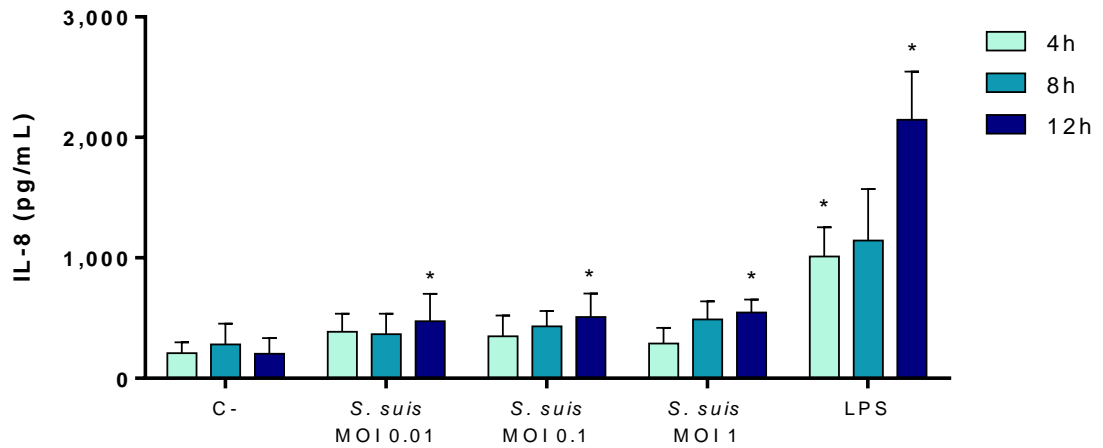**B****Cytotoxicity kinetics**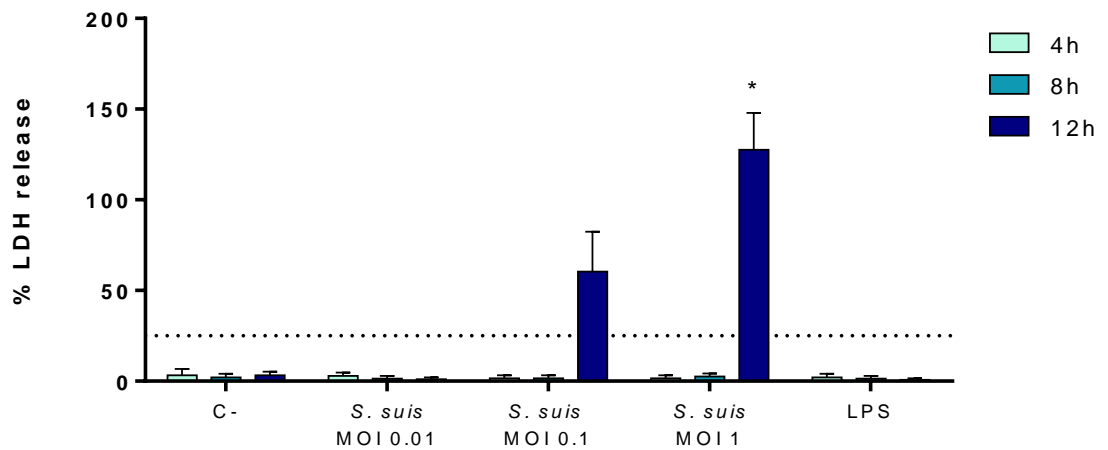

Supplement: S2 Fig — Purified porcine neutrophils were stimulated for different times with S. suis wild type at various multiplicity of infection (MOI) or with positive control lipopolysaccharide (LPS; 100 ng/mL). C- correspond to unstimulated control cells. (A) The IL-8 in the supernatant was quantified by ELISA. (B) Cytotoxicity was determined by measuring the amounts of lactate dehydrogenase (LDH) in fresh supernatant using a colorimetric reaction. The dotted line represents the threshold of 25% under which the condition is considered non-cytotoxic. * represents a significant difference compared to C- (P < 0.05). (PDF) [file pone.0296844.s002.pdf]

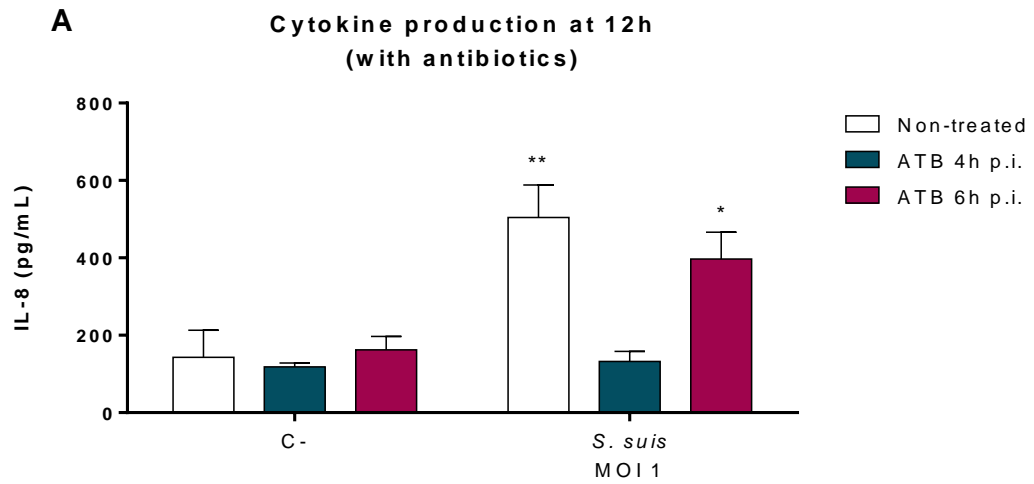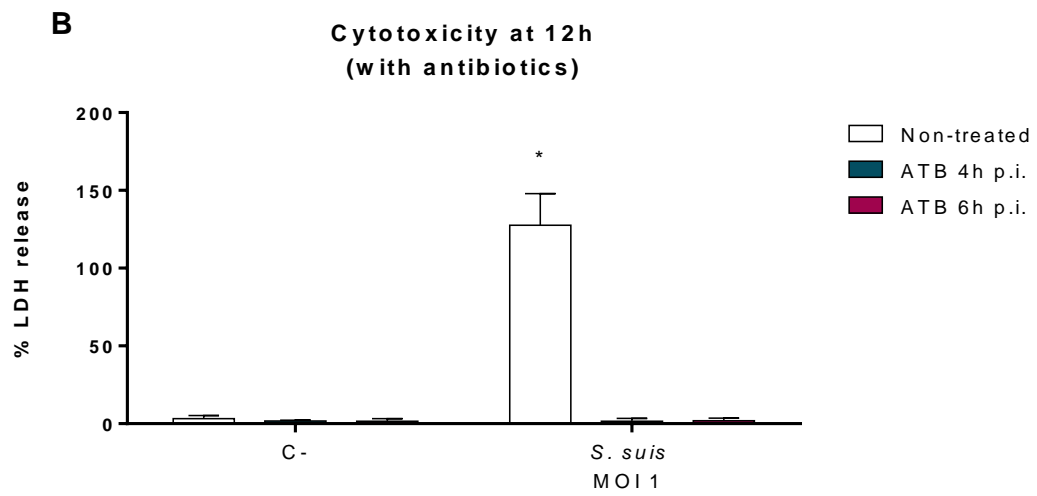

Supplement: S3 Fig — Purified porcine neutrophils were stimulated for a total of 12 h with S. suis wild type at a multiplicity of infection (MOI) of 1. After 4 h or 6 h post-infection (p.i.), 5 000 U/mL of antibiotics (ATB) were added to the cells to control bacteria multiplication. C- correspond to unstimulated control cells. (A) The IL-8 in the supernatant was quantified by ELISA. (B) Cytotoxicity was determined by measuring the amounts of lactate dehydrogenase (LDH) in fresh supernatant using a colorimetric reaction. The dotted line represents the threshold of 25% under which the condition is considered non-cytotoxic. * represents a significant difference compared to C- (P < 0.05). (PDF) [file pone.0296844.s003.pdf]

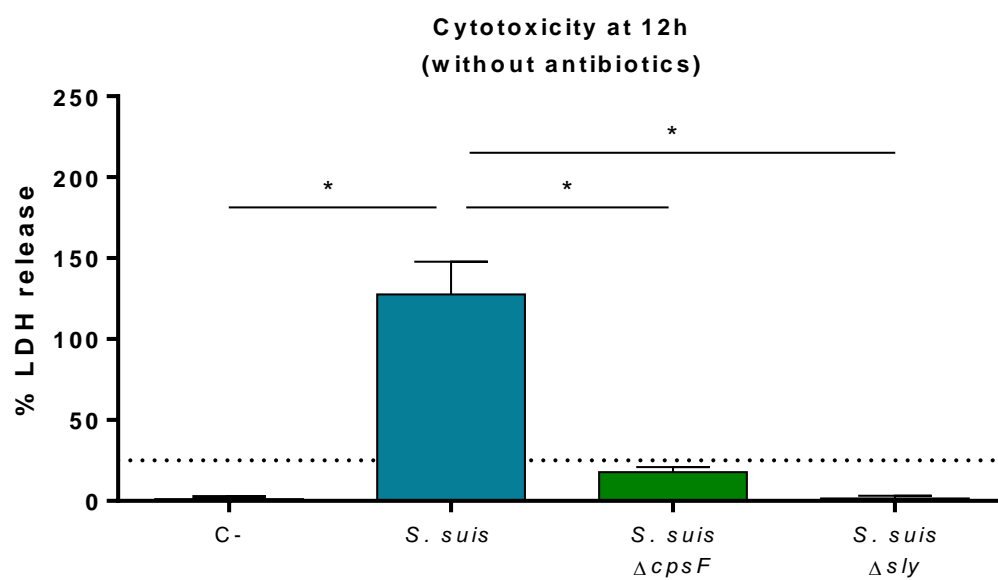

Supplement: S4 Fig — Purified porcine neutrophils were stimulated for 12 h with S. suis wild type, S. suis ΔcpsF, S. suis Δsly at a multiplicity of infection (MOI) of 1. After incubation, the supernatant was collected and the lactate dehydrogenase (LDH) measured by a colorimetric reaction. The LDH is released by lysed cells and its amount reflects the percentage of cytotoxicity. The dotted line represents the threshold of 25% under which the condition is considered non-cytotoxic. * represents a significant difference (P < 0.05). (PDF) [file pone.0296844.s004.pdf]

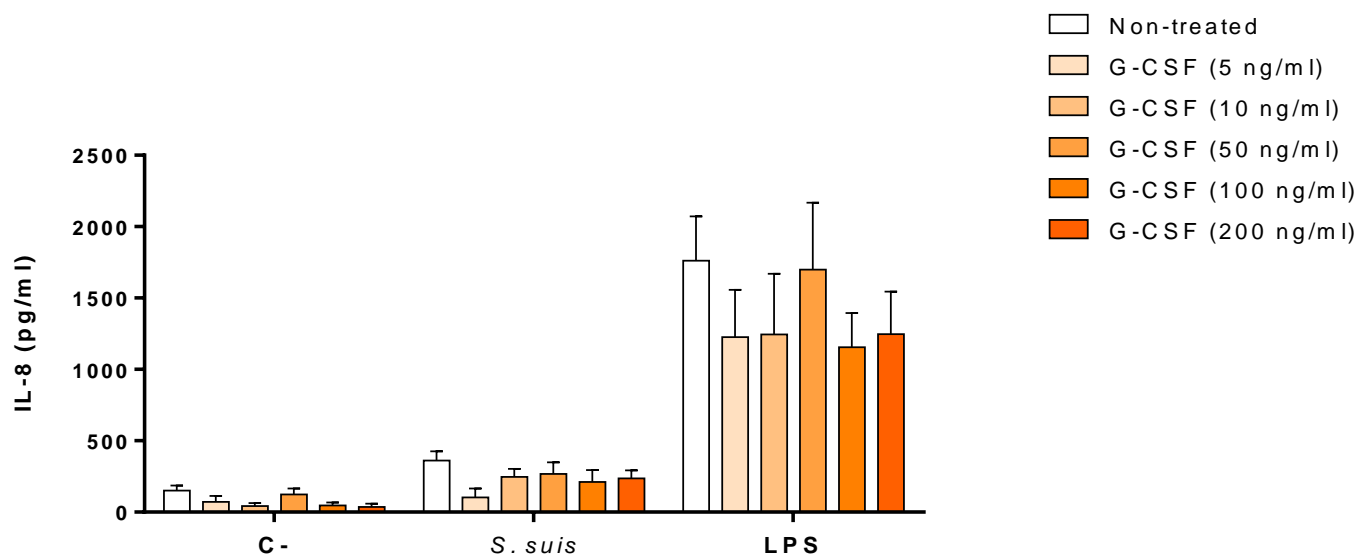

Supplement: S5 Fig — Porcine neutrophils were non-treated or primed with G-CSF (5, 10, 50, 100 or 200 ng/mL) for 30 min. They were then stimulated with S. suis at a multiplicity of infection (MOI) of 1; or positive control lipopolysaccharide (LPS; 100 ng/mL). C- correspond to unstimulated control cells. To prevent cytotoxicity caused by S. suis multiplication, antibiotics were added after 6 h of incubation. Neutrophils were stimulated 12 h and supernatant analysed by ELISA. Non-treated cells stimulated with S. suis and LPS produced higher IL-8 than unstimulated C- cells (P < 0.05 –not indicated). The statistical analyses were performed using the t-test or the Mann-Whitney rank sum test. (PDF) [file pone.0296844.s005.pdf]
